# Tracking antibiotic resistance in the environment: whole-genome sequencing of seven bacteria from the soil and water

**DOI:** 10.1128/mra.00098-25

**Published:** 2025-06-03

**Authors:** Danae K. R. Bardaji, Girish Kumar, Kamaria J. McFadden, Malik T. Jett, Kiyonna N. Jones, Malasia Berry, Ceonna Justin, Alexa Pedraza, André O. Hudson

**Affiliations:** 1 Thomas H. Gosnell School of Life Sciences, Rochester Institute of Technology 173222, Rochester, New York, USA; 2 Rochester Prep High School, Rochester, New York, USA; University of Maryland School of Medicine, Baltimore, Maryland, USA

**Keywords:** antibiotics, resistance, *Enterococcus hirae*, *Serratia marcescens*, *Bacillus amyloliquefaciens*, *Citrobacter braakii*, *Lactococcus garvieae*, *Enterococcus faecium*, *Lactococcus lactis*

## Abstract

Soil and water samples collected from the Rochester Institute of Technology campus were analyzed for antibiotic-resistant bacteria. Whole-genome sequencing and annotation were conducted on seven isolates that show a diverse range of resistance patterns, underscoring the urgent need for ongoing surveillance of antimicrobial resistance in the environment.

## ANNOUNCEMENT

Monitoring antibiotic-resistant bacteria in environmental settings is essential for understanding and mitigating the spread of resistance (1, 2). Antimicrobial resistance (AMR) is a pressing global health issue, threatening the efficacy of existing treatments and increasing the burden of infectious diseases (3). Advancements in genomic tools are crucial for tackling AMR, as they provide comprehensive insights into resistance mechanisms and aid in predicting emerging threats (4).

To investigate the presence of antibiotic-resistant bacteria in the environment, soil and water from a fountain were collected at the Rochester Institute of Technology (RIT), New York, USA. The samples were placed in sterile 50 mL conical tubes and incubated in tryptic soy broth (TSB) at 25°C with continuous shaking at 200 rpm for 7 days. Serial dilutions ranging from 10⁻¹ to 10⁻¹⁰ were prepared in TSB, and 100 µL of each dilution was plated on tryptic soy agar (TSA) plates, followed by incubation for 72 hours at 25°C. Bacterial colonies exhibiting distinct morphological characteristics (size, shape, color, and texture) were selected and cultured in TSB for 48 hours at 25°C with constant shaking at 150 rpm. Antibiotic susceptibility and resistance were assessed using a disk diffusion assay with commercial antibiotic disks, including rifampicin (5 mg/mL), polymyxin B (300 IU), vancomycin (30 mg/mL), chloramphenicol (30 mg/mL), colistin sulfate (10 mg/mL), sulfamethoxazole/trimethoprim (SXT) (25 mg/mL), and clindamycin (2 mg/mL), following the Clinical and Laboratory Standards Institute M100 guidelines (5). The antibiotic disks were applied to TSA plates inoculated with each isolate, followed by incubation for 48 hours at 25°C (6). After overnight incubation, the bacterial cultures were adjusted to a 0.5 McFarland standard and then swabbed onto the surface of the agar plates using a sterile swab before applying the antibiotic disks. Genomic DNA was isolated from 25 mL of TSB culture using the GenElute isolation kit (Sigma-Aldrich, USA), following the manufacturer’s instructions. DNA concentrations were measured using a Qubit 4.0 fluorometer with the 1× dsDNA High Sensitivity Kit (Life Technologies, MD, USA). Library preparation was done using the Nextera XT kit (Illumina Inc., USA), followed by sequencing on the Illumina MiSeq platform with a V3 Kit for 2 × 300 cycles at the RIT Genomics Lab.

Unless otherwise stated, all bioinformatics software was used with default parameters. Raw sequencing reads underwent quality control using fastp version 0.23.2 (7). High-quality reads were assembled using SPAdes version 3.15.4 (8), and the assembled genomes were taxonomically classified using JSpeciesWS (9). Genome annotation was conducted using the NCBI Prokaryotic Genome Annotation Pipeline (10). The assembly metrics and annotation details are provided in Table 1.

**TABLE 1:** Genome annotation information for the seven isolates

Sample	No. of raw reads	No. of nucleotides	SRA accession	Assembly accession	Species	Assembly size (bp)	Coverage (×)	No. of contigs	*N*_50_	GC (%)	No. of tRNA	No. of rRNA	No. of genes
RIT-850	8,146,610	2,269,270,254	SRX26938035	GCA_046765495.1	*Bacillus amyloliquefaciens*	3,911,483	582	19	2,052,448	45.8	72	3	4,079
RIT-851	4,129,204	1,182,150,429	SRX26938036	GCA_046765475.1	*Citrobacter braakii*	4,868,761	232	22	2,553,664	52.1	73	6	4,636
RIT-853	5,923,686	1,642,969,853	SRX26938037	GCA_046765455.1	*Enterococcus hirae*	2,965,036	513	31	203,590	36.6	56	3	2,741
RIT-854	4,517,072	1,238,054,692	SRX26938038	GCA_046765435.1	*Serratia marcescens*	5,169,575	243	54	511,440	59.9	81	7	4,982
RIT-855	5,900,326	1,649,537,537	SRX26938039	GCA_046765415.1	*Lactococcus garvieae*	2,232,496	717	51	239,227	37.9	48	4	2,284
RIT-857	3,819,416	1,089,939,891	SRX26938040	GCA_046765355.1	*Enterococcus faecium*	2,905,644	341	98	172,449	38.2	48	5	2,874
RIT-858	9,177,128	2,557,489,060	SRX26938041	GCA_046765335.1	*Lactococcus lactis*	2,471,492	1,112	25	344,694	34.8	48	4	2,420

**Fig 1:**
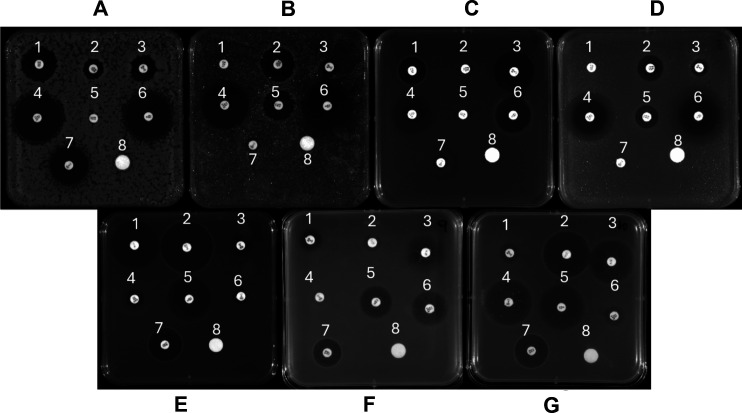
Antibiotic resistance profiles of the identified bacterial isolates: *Bacillus amyloliquefaciens* RIT 850 (**A**), *Citrobacter braakii* RIT 851 (**B**), *Enterococcus hirae* RIT 853 (**C**), *Serratia marcescens* RIT 854 (**D**), *Lactococcus garvieae* RIT 855 (**E**), *Enterococcus faecium* RIT 857 (**F**), and *Lactococcus lactis* RIT 858 (**G**). The tested antibiotics include (1) rifampicin (5 mg/mL), (2) polymyxin B (300 IU), (3) vancomycin (30 mg/mL), (4) chloramphenicol (30 mg/mL), (5) colistin sulfate (10 mg/mL), (6) SXT (25 mg/mL), (7) clindamycin (2 mg/mL), and (8) a negative control (10 µL of methanol).

Antibiotic resistance and susceptibility testing revealed varying resistance patterns based on the zones of inhibition or lack thereof. *Bacillus amyloliquefaciens* RIT 850 displayed resistance to colistin sulfate and intermediate susceptibility to polymyxin B and vancomycin (Fig. 1A). *Citrobacter braakii* RIT 851 exhibited resistance to rifampicin, vancomycin, and clindamycin (Fig. 1B). *Enterococcus hirae* RIT 853 showed resistance to polymyxin B and colistin sulfate (Fig. 1C). *Serratia marcesens* RIT 854 demonstrated intermediate susceptibility to vancomycin and colistin sulfate and resistance to rifampicin and clindamycin (Fig. 1D). *Lactococcus garvieae* RIT 855 was resistant to chloramphenicol and STX, with intermediate susceptibility to vancomycin (Fig. 1E). *Enterococcus faecium* RIT 857 exhibited resistance to polymyxin B and chloramphenicol (Fig. 1F), while *Lactococcus lactis* RIT 858 displayed intermediate susceptibility to rifampicin and STX (Fig. 1G). These findings highlight the diversity of antibiotic-resistant bacteria in the environment and emphasize the need for ongoing surveillance and genomic characterization of environmental AMR.

Whole-genome sequences of the seven bacterial isolates were analyzed using ResFinder to identify acquired antimicrobial resistance genes and chromosomal point mutations, applying a 90% identity threshold and a 60% minimum length for detection (11). The analysis revealed no resistance genes in *Lactococcus lactis* RIT858, *Lactococcus garvieae* RIT855, and *Bacillus amyloliquefaciens* RIT850. In contrast, *Enterococcus faecium* RIT857 harbored aac(6′)-li (98.18%), msr(C) (97.23%), and erm(B) (100%), while *Serratia marcescens* RIT854 carried aac(6′)-lc (97.28%), blaSRT-2 (96.83%), and tet(41) (99.15%). *Enterococcus hirae* RIT853 contained aac(6′)-lid (99.27%), tet(M) (96.46%), and tet(L) (100%), and *Citrobacter braakii* RIT851 possessed blaCMY-101 (99.04%).

## Data Availability

Table 1 presents the whole-genome assemblies, Sequence Read Archive (SRA), and accession numbers of the bacterial genomes, which can be downloaded from GenBank and SRA, respectively.
